# Reduced Vagal Compound Action Potential Amplitude Is Associated with Glomerular Injury in an Experimental Rabbit Model of Severe Subarachnoid Hemorrhage

**DOI:** 10.3390/jcm15156056

**Published:** 2026-08-04

**Authors:** Feyza Bayrakdar Çağlayan, Mehmet Emin Demir, Simge Bardak Demir, İskender Samet Daltaban, Mehmet Selim Gel, Mehmet Dumlu Aydın, Ayhan Kanat, Muhammet Enes Aydın, Elif Demirci, Siren Sezer

**Affiliations:** 1 Department of Nephrology, School of Medicine, Yüksek İhtisas University, Ankara 06530, Türkiye; 2 Department of Nephrology, School of Medicine, Atılım University, Ankara 06830, Türkiye; 3 Yenimahalle Education and Research Hospital, Ankara 06370, Türkiye; 4 A Life Hospital, Department of Neurosurgery, Ankara 06794, Türkiye; 5 Department of Neurosurgery, Faculty of Medicine, Karadeniz Technical University, Trabzon 61080, Türkiye; 6 Department of Neurosurgery, Faculty of Medicine, Atatürk University, Erzurum 25240, Türkiye; 7 Department of Neurosurgery, Faculty of Medicine, Recep Tayyip Erdoğan University, Rize 53100, Türkiye; 8 Department of Anesthesiology, Faculty of Medicine, Atatürk University, Erzurum 25240, Türkiye; 9 Department of Pathology, Faculty of Medicine, Atatürk University, Erzurum 25240, Türkiye

**Keywords:** subarachnoid hemorrhage, vagus nerve, renal innervation, autonomic dysfunction, neurogenic hypertension, acute kidney injury

## Abstract

**Background:** Subarachnoid hemorrhage (SAH) induces a sympathetic surge and systemic inflammation that may impair renal perfusion and glomerular integrity. Although the vagus nerve is central to autonomic and anti-inflammatory regulation, its relationship to renal structural injury in severe SAH is unclear. This study evaluated whether electrophysiological vagal CAP amplitude correlates with glomerular integrity in an experimental SAH model. **Methods:** Nineteen rabbits were assigned to control (*n* = 5), sham (*n* = 5), and SAH groups (*n* = 9). SAH was induced by daily cisterna magna injections of autologous blood for three days, and animals were followed for 14 days; those that did not survive to the 14-day endpoint formed the SAH-Nonsurvivor subgroup (*n* = 4). Vagal compound action potential (CAP) amplitude (mV) was recorded electrophysiologically and used as the principal physiologic readout of vagal nerve integrity. Renal tissue and perirenal parasympathetic ganglia were analyzed histologically and stereologically to quantify degenerated neurons and atrophic glomeruli (per mm^3^). Vagal CAP amplitude decreased from 1.42 ± 0.36 mV in controls to 0.34 ± 0.11 mV in the SAH-Nonsurvivor subgroup (*p* < 0.001), while atrophic glomeruli increased from 4 ± 1 to 98 ± 11 per mm^3^. Degenerated neuronal density peaked in the SAH-Nonsurvivor subgroup (98 ± 19 per mm^3^). Vagal CAP amplitude was inversely correlated with glomerular injury (Spearman ρ = −0.89; 95% CI −0.96 to −0.73; *p* < 0.001). **Conclusions:** In this small exploratory study, reduced vagal CAP amplitude was associated with greater glomerular injury after severe SAH. These hypothesis-generating findings warrant confirmation in larger, adequately powered studies before any physiologic or translational interpretation can be made.

## 1. Introduction

Subarachnoid hemorrhage (SAH) is a life-threatening condition frequently accompanied by systemic complications caused by excessive sympathetic activation and inflammation [1,2]. The resulting presumed catecholamine surge may contribute to widespread vasoconstriction, hypertension, and ischemic damage in peripheral organs, including the kidneys [2,3]. Acute kidney injury (AKI) develops in up to 40% of SAH patients and is strongly associated with elevated inflammatory biomarkers and poor outcomes [2,3,4,5].

The kidney receives dense sympathetic innervation, whereas its parasympathetic supply is more limited and conceptually distinct from the systemic cholinergic anti-inflammatory pathway (CAIP). The CAIP is mediated by efferent vagal signaling to the celiac ganglion, which in turn modulates the splenic sympathetic nerve and acetylcholine-releasing T cells, ultimately attenuating macrophage-derived cytokines such as TNF-α [6,7]. In contrast, the kidney itself receives a sparser direct parasympathetic input via perirenal/renal hilar microganglia, the functional role of which remains incompletely characterized [8]. These two pathways (systemic CAIP and intrinsic renal parasympathetic innervation) are anatomically and mechanistically distinct. In the present study, “vagal function” refers to the electrophysiological integrity of the cervical vagal trunk and the histologic preservation of perirenal parasympathetic ganglia, rather than to systemic anti-inflammatory tone, which we did not directly measure.

In this manuscript, “loss of vagal function” refers specifically to a reduction in evoked vagal compound action potential amplitude accompanied by histologic degeneration of vagal axons and perirenal ganglion neurons—that is, a structural/electrophysiological deficit rather than a transient autonomic tone shift. Such vagal dysfunction has been described in several clinically relevant conditions, including aneurysmal subarachnoid hemorrhage and traumatic brain injury (where brainstem and nucleus tractus solitarius injury impairs efferent vagal output) [9,10], advanced chronic kidney disease (where uremic neuropathy reduces vagal nerve activity) [6], diabetic autonomic neuropathy, and sepsis-related autonomic failure. In these settings, reduced vagal signaling may be compatible with sympathetic predominance, renal vasoconstriction, and glomerular ischemia [10,11,12].

Previous SAH models demonstrated degeneration of vagal ganglia and renal artery vasospasm, suggesting a neurogenic mechanism of kidney injury, but no study has established a direct functional link between vagal CAP amplitude and glomerular preservation [8]. In severe SAH, this autonomic imbalance may accompany peripheral organ injury, including the kidney [13,14]. While vagus nerve stimulation has been shown to improve renal allograft function in experimental transplantation, the direct relationship between vagal nerve function and glomerular preservation remains unclear.

The conceptual framework of this study is as follows. Severe SAH produces autonomic dysregulation in which sympathetic overactivity is normally opposed by vagal (parasympathetic) tone; structural injury to vagal pathways may therefore be compatible with reduced counter-regulatory signaling and, together with hypertension and inflammation, may accompany peripheral organ injury. Within this framework, the cervical-vagal CAP amplitude provides a measurable index of vagal structural integrity, and atrophic glomerular density provides a measurable index of renal structural injury. We use these two quantifiable endpoints to test, at the level of statistical association, whether vagal structural integrity tracks with renal structural injury after severe SAH. This study aimed to determine whether vagal CAP amplitude is associated with renal structural injury in an experimental rabbit model of severe SAH. We tested the hypothesis that lower vagal CAP amplitude is associated with greater glomerular injury. We emphasize at the outset that this is an exploratory, association-level analysis in a small cohort: the design cannot establish causality, and the model does not reproduce the formal clinical diagnosis of brain death (which requires demonstration of irreversible loss of all brainstem function, including standardized apnea testing). We therefore describe outcomes in terms of survival and SAH severity rather than brain death.

## 2. Materials and Methods

Experimental Design and Animal Model: This study was conducted in full compliance with National Institutes of Health animal research guidelines and received approval from the Atatürk University Ethics Committee. Nineteen New Zealand white rabbits (2.5–3.0 kg) were randomly assigned to one of three groups: Control (*n* = 5), Sham (*n* = 5), and SAH (*n* = 9). Animals were initially randomized into these three experimental groups; for outcome-based analyses, the SAH group was subsequently stratified post hoc into SAH-Survived and SAH-Nonsurvivor subgroups according to whether terminal brainstem failure occurred before the 14-day endpoint.

All animals were housed under standard conditions with free access to food and water. Adult male New Zealand white rabbits (age 12–16 weeks) from a single accredited supplier were used; only male animals were studied to limit hormonal variability, which is acknowledged as a limitation. Baseline body weights were comparable across groups (Control 2.8  ±  0.2 kg, Sham 2.7  ±  0.2 kg, SAH 2.8  ±  0.3 kg; not statistically different). Over follow-up, Control and Sham animals maintained or slightly increased body weight, SAH-Survived animals lost approximately 12–18% of baseline weight, and SAH-Nonsurvivor animals showed the greatest weight loss (>20%) in the days preceding death, consistent with their higher composite clinical scores. Allocation was performed by an investigator not involved in subsequent outcome assessment using sequentially generated random numbers (random.org), with allocation concealed in sealed opaque envelopes opened immediately before the first procedure. Pre-defined exclusion criteria were peri-operative anesthetic death (<30 min from induction) and failure of cisterna magna cannulation; no animals were excluded under these criteria. Humane endpoints were pre-specified as loss of righting reflex with respiratory arrest unresponsive to stimulation, or >20% body-weight loss with score ≥ 10 on the composite clinical score. Buprenorphine (0.05 mg/kg, s.c.) was administered every 12 h for 48 h after each cisterna magna procedure and additional analgesia given on welfare grounds. Mechanical ventilation was not used outside of brief terminal resuscitation attempts. All histopathologic, stereologic, electrophysiologic, and image-based analyses were performed by investigators blinded to group assignment; sample identifiers were re-coded by a third party before analysis. To assess reproducibility, a random subset of approximately 20% of sections was independently re-counted by a second blinded observer and re-counted by the primary observer on a separate occasion; inter-observer and intra-observer agreement for glomerular and neuronal counts were high (intraclass correlation coefficients above 0.90), and the mean of replicate counts was used for analysis. No formal a priori power calculation was performed. This study is explicitly exploratory and pilot in nature. However, the small per-group sample size (*n* = 4–5) limits statistical power, precludes robust multivariable modeling, and means that all effect estimates carry wide uncertainty. The sample size was based on minimum-feasible numbers consistent with the 3Rs principle and with previously published rabbit SAH models. We do not present any finding as confirmatory. Both kidneys were harvested; reported glomerular and arterial morphometric values represent the mean of left and right kidneys for each animal.

SAH was induced by serial cisterna magna injections of autologous arterial blood (0.5 mL/day for 3 consecutive days), a well-established model of increased intracranial pressure and progressive brain injury. Under general anesthesia (ketamine 35 mg/kg and xylazine 5 mg/kg, intramuscular), each rabbit in this group received a cisterna magna injection of 0.5 mL fresh non-heparinized arterial blood (drawn from the auricular artery). This injection was performed once daily on three consecutive days (total of ~1.5 mL of blood introduced). This protocol is known to elevate intracranial pressure and produce diffuse SAH, leading to brainstem ischemia over time. Sham group rabbits underwent identical anesthesia, positioning, and surgical exposure of the cisterna magna as the SAH group, but received 0.5 mL of pre-warmed, sterile 0.9% saline injected slowly (over approximately 30 s) into the cisterna magna on three consecutive days (total volume administered: 1.5 mL over 3 days), exactly matching the volume, frequency, and administration duration of the SAH protocol. Control rabbits underwent no injections or surgical intervention.

All animals were monitored for 14 days for neurological signs of brainstem dysfunction, cardiorespiratory instability, and survival status. Non-invasive tail-cuff blood pressure (BP) was recorded each morning during a 1-min monitoring window (average of three consecutive cuff measurements), and daily mean BP values were retained for longitudinal analysis (Supplementary Figure S3). Severity of illness in SAH animals at the 14-day end-point was scored using a composite clinical score (0–3 each for activity, posture, feeding, and respiratory pattern; maximum 12); SAH-Survived animals had end-point scores of 6–9, with persistently reduced activity, transient apneic episodes, and weight loss of 12–18% from baseline, whereas SAH-Nonsurvivor animals had scores ≥10 in the 24–48 h preceding death.

*Physiological Monitoring:* Physiological parameters, including peripheral oxygen saturation (SpO_2_), pulse rate, systolic and diastolic blood pressures, and respiratory rate, were monitored non-invasively at baseline before the first injection and during daily monitoring sessions throughout the 14-day observation period. Oxygen saturation and pulse rate were measured using a handheld veterinary pulse oximeter (PalmSAT 2500A VET, Nonin Medical Inc., Plymouth, MN, USA). A veterinary-specific micro-clip probe was gently attached to a vascular, non-pigmented area on the inner surface of the auricular pinna while avoiding compression of the central auricular artery. Before probe placement, the fur at the measurement site was gently clipped and the skin was cleaned to optimize light transmission.

Systolic and diastolic blood pressures were measured using a small-animal tail non-invasive blood pressure amplifier (NIBP200A, BIOPAC Systems Inc., Goleta, CA, USA) with a compatible tail occlusion cuff and pulse sensor positioned around the proximal portion of the tail. Before each measurement session, the tail was gently warmed using a localized heating pad to reduce peripheral vasoconstriction and improve caudal arterial blood flow. Respiratory movements were recorded using a respiratory effort transducer (SS5LB, BIOPAC Systems Inc., Goleta, CA, USA) positioned snugly around the chest. The blood pressure and respiratory signals were acquired through a multichannel data acquisition system (MP100A-CE, BIOPAC Systems Inc.) interfaced with a universal interface module (UIM100C), sampled at 500 Hz, and processed using AcqKnowledge software (BIOPAC Systems Inc.). Measurements were accepted after stable signals had been obtained and the pulse rate displayed by the pulse oximeter was consistent with the heart rate determined by cardiac auscultation. Three consecutive stable measurements were obtained during each monitoring session and averaged for analysis. Clinical signs of brainstem dysfunction, including changes in respiratory pattern, loss of cranial nerve reflexes, and coma, were documented concurrently.

We defined the pre-specified moribund/terminal clinical state in this model by the occurrence of apnea or agonal respiration with a loss of cranial nerve reflexes, accompanied by a precipitous blood pressure rise followed by hypotension and cardiac arrest (if it occurred). Animals that met the pre-specified humane endpoint criteria, consistent with terminal brainstem dysfunction or moribund status, were euthanized immediately and included in the analysis as described below.

*Tissue Harvesting and Vagal Compound Action Potential (CAP) Amplitude Measurement:* At the end of the 14-day period, all surviving rabbits were deeply anesthetized (sodium pentobarbital 50 mg/kg i.v.) and then euthanized by decapitation. For rabbits that died or were euthanized early due to severe illness (particularly in the SAH group), data up to the point of death were included, and tissues were collected as soon as possible post-mortem. Specifically, in all SAH-Nonsurvivor animals, the interval between cessation of spontaneous circulation (defined by loss of palpable femoral pulse and flat-line ECG) and initiation of cervical vagal nerve dissection plus ipsilateral nephrectomy was recorded and was kept below 30 min (median 18 min, range 5–30 min). Tissues were immediately placed in ice-cold normal saline during dissection and transferred to 10% neutral buffered formalin within a further 5–10 min to minimize autolytic change. We thus had four experimental cohorts for analysis based on actual outcomes: Control (survived, no SAH), Sham (survived, saline only), SAH-Survived (SAH group animals that survived to 14 days, *n* = 5), and SAH-Nonsurvivor (SAH group animals that succumbed before day 14, *n* = 4; these reached the pre-specified moribund/terminal state defined above and were found moribund or in respiratory arrest despite brief attempts at supportive resuscitation).

*Vagal Compound Action Potential (CAP) Amplitude:* Immediately after sacrifice, we surgically exposed the cervical vagi and the upper thoracic vagal nerve trunks. We measured the compound action potential amplitude of the vagus nerve complex in each animal. A bipolar stimulating electrode was placed on the cervical vagus nerve, and a recording electrode was placed distally on the vagal trunk. A supramaximal electrical stimulus (0.1 ms pulses, at an intensity individually set at approximately 20% above the level producing a maximal response; the value shown on the stimulator [Students Stimulator SS1249, C. F. Palmer (London) Ltd., London, UK] display in the Figure 1 schematic, 10 mV, is one representative setting and not a fixed intensity used in all animals) was delivered. The evoked neural voltage signals were initially routed through an Isolated Preamplifier (Model 50-5966, Harvard Apparatus, Holliston, MA, USA) and subsequently through a dedicated electroencephalogram and microvolt amplifier module (EEG100C, BIOPAC Systems Inc., Goleta, CA, USA) connected to the primary data acquisition unit (MP100A-CE, BIOPAC Systems Inc.). The signals were band-pass filtered at 10–3 kHz within the amplifier system, displayed on an oscilloscope, and digitally recorded using AcqKnowledge software (BIOPAC Systems Inc.) for peak-to-peak amplitude analysis. Bipolar recording electrodes were separated by a fixed inter-electrode distance of approximately 5 mm, and the exposed nerve was kept moist with warm 37 °C saline to maintain tissue temperature. To ensure comparability, vagal CAP recordings were obtained under identical stimulation and recording conditions in all four cohorts and immediately after sacrifice; for early-death (SAH-Nonsurvivor) animals, the identical protocol was applied as soon as possible post-mortem (median 18 min, range 5–30 min), and this timing difference is explicitly considered as a limitation below. Three consecutive evoked responses were averaged per animal; no additional signal-averaging or artifact-rejection procedures beyond this triplicate averaging were applied. The peak-to-peak amplitude (in millivolts, mV) of the vagal nerve response was taken as the vagal CAP amplitude value. This electrophysiological compound action potential amplitude reflects functional integrity of vagal fibers—higher amplitudes indicate preserved neural transmission, whereas lower amplitudes suggest severe vagal injury. Three measurements were taken and averaged per animal.

Following electrophysiological measurements, we harvested the kidneys and associated autonomic ganglia. Specifically, we carefully dissected the renal hila to identify the small parasympathetic ganglia in the renal plexus (hereafter referred to as renal parasympathetic ganglia or perirenal vagal ganglia). These ganglia, also known as renal hilar microganglia, receive vagal input and lie along the renal arteries. Both left and right kidneys along with adjacent renal plexus tissue were removed. Tissue samples (vagal nerve roots, renal ganglia, and kidneys) were fixed in 10% neutral buffered formalin for 7 days.

*Histopathological Analysis and Stereological Quantification:* Fixed tissues were processed by standard paraffin embedding. Serial 5 μm sections were obtained from each vagal nerve root (at the level of the nodose ganglion and cervical trunk), each renal ganglion, and the renal cortices. Sections were stained with hematoxylin and eosin (H&E) for general histopathology. Additional sections were processed with TUNEL staining (for apoptosis) and neuron-specific enolase (NSE) immunohistochemistry to aid identification of neurons and degenerative changes.

We evaluated vagal nerve pathology for evidence of Wallerian degeneration (axon fragmentation, myelin debris) and renal ganglion pathology for neuronal degeneration (pyknotic nuclei, cytoplasmic eosinophilia, cell dropout). Degenerated neurons in the renal parasympathetic ganglia were identified by criteria including cell shrinkage, dark eosinophilic cytoplasm, nuclear condensation, and perineuronal vacuolation. Only neurons positive for NSE (Neuron Spesific Enolase) were counted, and those meeting degeneration criteria were tallied as “degenerated neurons.”

For the kidneys, we examined both gross morphology and microscopic changes. Particular attention was paid to the renal arteries for any vasospasm or wall thickening, evidence of intrarenal hemorrhages, and the condition of glomeruli. Atrophic glomeruli were defined histologically by collapse of capillary tufts, wrinkling of the glomerular basement membrane, and diminished glomerular tuft size with sclerosis, often accompanied by peri-glomerular fibrosis. Normal glomeruli, in contrast, had open capillary loops and normal cellularity.

We employed stereological methods to quantify glomerular numbers and densities. Using the optical disector and Cavalieri method, every 10th section through the kidney was selected to ensure a systematic random sample of approximately 100 sections per kidney. On each sampled section, a counting frame grid was overlaid on digital photomicrographs. Glomerular profiles were counted in disector pairs of consecutive sections: a glomerulus counted in the reference section but not in the lookup section was considered one counted object (Sigma Q minus). Using the disector formula Nv = Sigma Q minus divided by (t multiplied by A frame), we calculated the numerical density of glomeruli (Nv, glomeruli per cubic millimeter) for each kidney. Here, t represents section thickness (5 micrometers), and A frame is the area of the counting frame. Atrophic and nonatrophic glomeruli were distinguished based on the histologic criteria described above. The density of atrophic glomeruli (per cubic millimeter) was obtained for each kidney. We also estimated the total number of glomeruli per kidney by multiplying the glomerular density by the kidney reference volume, determined using the Cavalieri principle from sectioned areas and section thickness.

Finally, the density of degenerated neurons in the renal ganglia was determined by counting degenerated neuron profiles in serial ganglion sections using a similar optical fractionator approach. Neuronal density was expressed as degenerated neurons per cubic millimeter of ganglion volume.

### Statistical Analysis

All data were analyzed using IBM SPSS Statistics (v20.0, Chicago, IL, USA). Continuous variables are presented as mean ± standard deviation (SD). Data distribution was tested using the Shapiro–Wilk test. For normally distributed data (e.g., blood pressure, vagal CAP amplitude), comparisons across the four analytical cohorts (Control, Sham, SAH-Survived, SAH-Nonsurvivor) were made with one-way analysis of variance (ANOVA), followed by Tukey’s post hoc test. For non-parametric data or non-normally distributed outcomes (e.g., histological counts), we used the Kruskal–Wallis test with Dunn’s post hoc comparisons. Given the small per-group sample sizes (*n* = 4–5), normality testing has limited power; therefore, Kruskal–Wallis with Dunn’s post hoc was the primary approach for all histologic counts and continuous outcomes with skewed distributions, and ANOVA results are reported only where parametric assumptions were reasonable. An exploratory multivariable linear regression was also run; because the per-predictor case ratio is far below the threshold recommended for stable estimation, it is reported only in the Supplementary Material (Supplementary Table S1) and is not interpreted in the main text. The correlation between vagal CAP amplitude and glomerular counts was evaluated using Pearson’s correlation coefficient (for parametric data) or Spearman’s rho (for non-parametric data). Survival distributions across groups were compared using the Kaplan–Meier method with the log-rank test. Quantitative immunohistochemistry (TUNEL, NSE) and stereologic data (atrophic glomeruli and degenerated neurons per mm^3^) were compared between groups using Kruskal–Wallis tests with Dunn’s post hoc comparisons. Correlations between vagal CAP amplitude and renal/morphometric parameters were displayed as scatterplots with regression lines and 95% confidence intervals. Because the SAH-Survived and SAH-Nonsurvivor subgroups were defined post hoc according to 14-day survival status, all subgroup-level analyses (i.e., those that compare SAH-Survived with SAH-Nonsurvivor) are explicitly interpreted as exploratory. A two-tailed *p* value < 0.05 was considered statistically significant. Given the exploratory nature of the study and the small sample size, group-wise comparisons were interpreted primarily in terms of effect size and consistency across related endpoints rather than as confirmatory hypothesis tests; Dunn’s post hoc procedure incorporates a built-in adjustment for multiple pairwise comparisons and was applied consistently to all Kruskal–Wallis analyses, and we did not perform additional families of unplanned comparisons. Because of the limited sample size and the resulting risk of multiplicity, we did not treat any single pairwise *p*-value as independently confirmatory. For the primary correlation, the Spearman coefficient is reported with its bootstrap 95% confidence interval (rho = −0.89, 95% CI −0.96 to −0.73; 2000 resamples). With only 19 observations for two predictors, residual diagnostics, variance-inflation assessment, the coefficient of determination, and coefficient confidence intervals would all be unstable and were therefore not computed. For this reason the regression has been moved out of the main text to Supplementary Table S1, where it is presented as a sensitivity analysis only; its coefficients are not treated as effect estimates and are not used to support any statement in this manuscript.

## 3. Results

### 3.1. Survival, Clinical Course, and Physiological Findings

All control and sham-operated rabbits remained alive and neurologically normal through the 14-day experiment. In contrast, the SAH group exhibited high mortality and distinct signs of severe brainstem dysfunction.

*Mortality:* Out of 9 SAH-induced rabbits, 4 animals (44%) died or had to be euthanized due to the pre-specified moribund/terminal clinical state before day 14 (the “SAH-Nonsurvivor” subgroup, *n* = 4). The remaining 5 SAH animals survived to 14 days (the “SAH-Survived” subgroup). We observed that the rabbits which succumbed tended to do so in the second week post-SAH (days 8–14). Two SAH group rabbits died on day 10, one on day 12, and one on day 14 with agonal breathing despite supportive care. No deaths occurred in the control or sham groups. Kaplan–Meier survival analysis with the log-rank test confirmed significantly lower survival in the SAH group compared with controls and sham (log-rank *p* = 0.015; 14-day cumulative survival: control 100%, sham 100%, SAH 56% [95% CI 21–86%]) (Supplementary Figure S1).

*Blood Pressure and Heart Rate:* Baseline mean arterial pressures (MAP) were similar across groups (95–105 mmHg) prior to interventions (Supplementary Figure S3). By the end of the experiment, clear differences emerged. The Control group maintained a near-constant MAP of 99 ± 7 mmHg. Sham animals had a mild, non-significant increase to 106 ± 8 mmHg. The SAH group, however, developed sustained elevations in mean arterial pressure (i.e., daily MAP readings consistently above baseline values, rather than isolated transient peaks). SAH-Survived rabbits had an average MAP of 117 ± 13 mmHg (significantly higher than control, *p* < 0.05). The SAH-Nonsurvivor subgroup exhibited more marked elevations in blood pressure prior to death: their average MAP exceeded 126 ± 8 mmHg, with peak readings (defined as the maximum single non-invasive tail-cuff systolic measurement obtained during a 1-min monitoring window) exceeding 135 mmHg. This was often accompanied by a terminal drop in pressure just before cardiac arrest. Heart rate (HR) showed biphasic changes in SAH animals. In the early phase of SAH (first 24–48 h after blood injection), SAH rabbits developed bradycardia (mean HR 156 ± 12 beats/min) compared to controls (252 ± 31 beats/min) and shams, consistent with the Cushing response. In the late phase of SAH (days 3–7), surviving SAH animals developed significant tachycardia (mean HR 349 ± 31 beats/min by day 7) along with episodic blood pressure surges. These tachycardic episodes corresponded to sympathetic hyperactivity following presumed brainstem failure.

Control and sham animals remained neurologically intact (normal appetite, activity, and reflexes) and maintained stable respiration (about 24 ± 4 breaths per minute, oxygen saturation about 96%). In contrast, SAH animals showed signs of brainstem dysfunction. Early after SAH induction, the respiratory rate dropped (to about 13 ± 3 breaths per minute) and oxygen saturation decreased (83 ± 10%), indicating neurogenic respiratory depression. As brain herniation progressed, irregular breathing patterns appeared, characterized by episodes of apnea alternating with rapid shallow breaths (tachypnea up to 40–45 breaths per minute). In moribund SAH rabbits, we observed gasping respirations, diaphragmatic breathing without chest expansion, and ultimately respiratory arrest. All SAH-Nonsurvivor rabbits experienced respiratory failure as the immediate cause of death. Pupillary reflexes were absent in these animals, consistent with severe terminal brainstem dysfunction in this model. We did not perform standardized apnea testing or the full ancillary work-up required to diagnose brain death, and we therefore do not claim that these animals met formal brain-death criteria.

*ECG changes:* SAH also provoked cardiac electrical disturbances. In SAH rabbits (especially those that died), we documented ST-segment depressions, frequent ventricular ectopic beats including bigeminy, sporadic ventricular tachyarrhythmias, and episodic atrioventricular conduction block on ECG. These arrhythmias reflected the cardiac effects of massive sympathetic discharge and intracranial pressure fluctuations.

Neurogenic pulmonary edema (NPE) developed in the severe SAH cases. On necropsy, the lungs of SAH-Nonsurvivor rabbits were heavy, wet, and showed frothy fluid in airways. Histologically, they had pulmonary capillary congestion and intra-alveolar edema with proteinaceous fluid—classic signs of NPE. All SAH-Nonsurvivor rabbits with NPE also had extensive renal injury (as detailed below), suggesting a common underlying mechanism of autonomic dysfunction and presumed sympathetic overactivation, although direct catecholamine measurements were not performed.

*Vagal CAP Amplitude Outcomes:* Vagal CAP amplitude declined progressively with increasing severity of SAH injury. Figure 1 illustrates the experimental setup for vagal nerve stimulation and recording. In healthy control rabbits, the vagus nerve exhibited a robust compound action potential with an average amplitude of 1.42 ± 0.36 mV. Sham-operated rabbits had a slightly lower vagal response (1.23 ± 0.29 mV), but this was not significantly different from controls (*p* > 0.1), indicating that the surgical procedure and saline injection had minimal effect on vagal function. Group-wise hemodynamic, vagal CAP amplitude, and stereologic kidney data are summarized in Table 1.

In contrast, rabbits subjected to SAH showed significantly reduced vagal CAP amplitude. SAH-Survived animals (those that exhibited progressive brainstem dysfunction but survived to the 14-day endpoint) had a mean vagal amplitude of 0.95 ± 0.12 mV, approximately a 33% reduction versus controls (*p* < 0.01). More strikingly, the SAH-Nonsurvivor subgroup (those that experienced fatal SAH with terminal brainstem dysfunction) had an average vagal CAP amplitude of only 0.34 ± 0.11 mV. This represents a 75% reduction from control values (*p* < 0.001). The difference in vagal CAP amplitude between SAH-Nonsurvivor and SAH-Survived animals was also significant (*p* < 0.01), indicating that vagal nerve function was worse in the animals with the most severe outcomes.

In all rabbits that died, vagal CAP amplitude at endpoint was < 0.5 mV. In fact, just before death, two rabbits had nearly flat vagal traces (~0.1–0.2 mV), suggesting almost complete vagal nerve failure. In contrast, rabbits that survived to the 14-day endpoint maintained vagal CAP amplitudes near 1.0 mV. Thus, in this dataset low vagal CAP amplitudes were associated with progression to terminal brainstem failure. Because vagal CAP amplitude was recorded immediately after sacrifice (or, in early-death animals, within 30 min of cardiac arrest; see Methods), we cannot fully exclude that the somewhat lower amplitudes in the SAH-Nonsurvivor subgroup were partly influenced by a longer post-mortem interval. Electrophysiologic responses were undetectable in two SAH-Nonsurvivor animals in which the post-mortem interval was minimal; however, the stability of vagal CAP amplitude across the 5–30 min post-mortem window was not validated in this study, and no such validation experiment was performed. A contribution of post-mortem tissue deterioration to the reduced amplitudes recorded in this subgroup therefore cannot be excluded, and the SAH-Nonsurvivor values should be read as potentially biased by differential recording conditions (see Limitations).

### 3.2. Histopathological Findings in Vagal Ganglia and Kidneys

*Renal Parasympathetic Ganglia Degeneration:* Histological examination of the renal hilar (perirenal) ganglia revealed minimal abnormalities in control and sham animals. These ganglia in control rabbits contained healthy neurons with visible Nissl substance and only rare apoptotic cells. The average density of degenerated neurons in control ganglia was 6 ± 2 per cubic millimeter, indicating negligible neurodegeneration. Sham ganglia showed a slight increase in degenerative changes (21 ± 5 degenerated neurons per cubic millimeter), possibly due to surgical stress or local inflammation, but the overall ganglion architecture remained intact.

*SAH Animals:* SAH animals demonstrated markedly higher degeneration in the renal parasympathetic ganglia. In SAH-Survived rabbits, the ganglia contained shrunken, hyperchromatic neurons with perineuronal halos, consistent with ischemic degeneration. The degenerated neuron density averaged 25 ± 7 per cubic millimeter, which was significantly greater than in the sham group (*p* < 0.05). Some neurons were TUNEL positive, indicating apoptotic cell death, and occasional neuronophagia, defined as microglial ingestion of degenerating neurons, was observed. Quantitatively, TUNEL-positive neurons per ganglion section averaged 2.1 ± 0.8 in controls, 4.5 ± 1.2 in sham, 9.8 ± 2.4 in SAH-Survived, and 38.7 ± 7.6 in SAH-Nonsurvivor animals (Kruskal–Wallis *p* < 0.001; Dunn’s post hoc: SAH-Nonsurvivor vs. control *p* < 0.001); Supplementary Figure S2 summarizes the stepwise increase in TUNEL-positive neurons per ganglion section across groups; NSE immunostaining was used to confirm neuronal identity for counting and followed the same qualitative pattern.

*SAH-Nonsurvivor Subgroup:* In the SAH-Nonsurvivor subgroup, ganglionic damage was extreme. The ganglia were often difficult to identify macroscopically, appearing pale and fibrotic. Microscopically, these ganglia had lost the majority of their neurons; the few remaining cells appeared as ghost neurons, showing only faint outlines or fragmented DNA on TUNEL staining. The density of degenerated neurons in SAH-Nonsurvivor rabbits reached 98 ± 19 per cubic millimeter, approximately four times higher than in the SAH-Survived animals (*p* < 0.01). Essentially, almost all neurons in the renal vagal ganglia exhibited degenerative changes in the animals with the most severe (fatal) SAH outcomes. Degenerated neuron density thus increased in parallel with SAH severity. We note that sympathetic activation was inferred from hemodynamic signs rather than measured, so any link to ganglion neuron loss is hypothetical.

Glomerular Pathology: The glomeruli were the main focus of our analysis as an endpoint of renal injury. In control rabbits, renal glomeruli were uniformly normal, showing well-perfused capillary loops, normal cellularity, and no evidence of sclerosis or collapse. Control kidneys had only a few atrophic glomeruli (typically fewer than five per entire kidney section), often located peripherally and likely representing incidental age-related changes. The density of atrophic glomeruli in controls was 4 ± 1 per cubic millimeter, indicating that more than 95% of glomeruli were healthy (representative normal and atrophic glomeruli are shown in Figure 2). Sham animals showed a mild increase in atrophic or ischemic glomeruli (12 ± 3 per cubic millimeter), but the vast majority of glomeruli remained normal in appearance. There were no significant differences between control and sham groups in total glomerular counts or kidney mass, suggesting that the sham procedure itself did not cause major renal injury.

SAH had a dramatic impact on glomeruli. Figure 3 illustrates normal and atrophic glomeruli in an SAH rabbit kidney. In SAH-Survived rabbits, many glomeruli, especially in the subcapsular region, showed partial collapse and contraction. These animals had an atrophic glomerulus density of 56 ± 8 per cubic millimeter, accounting for roughly half of all glomeruli in a given volume. The remaining glomeruli in these rabbits, while not fully atrophic, often displayed signs of injury such as mesangial expansion or early fibrosis. Stereological analysis revealed that the total number of normal glomeruli per kidney was significantly reduced in SAH-Survived animals compared with controls (approximately 40% fewer normal glomeruli, *p* < 0.01). Group-wise atrophic glomerular density is shown as a box-and-whisker plot in Supplementary Figure S4A, and the scatterplot of vagal CAP amplitude versus atrophic glomerular density with the fitted regression line and 95% confidence interval is shown in Supplementary Figure S4B.

*Renal Vascular Changes:* We found that severe SAH was accompanied by renal artery vasospasm and damage to intrarenal vessels. In SAH-Nonsurvivor rabbits, the main renal arteries often exhibited pronounced vasoconstriction, with narrowed lumens and thickened smooth muscle layers. Quantitatively, on H&E-stained transverse sections of the proximal renal artery (analyzed in ImageJ by a blinded observer; mean of three sections per animal), luminal diameter was 0.86 ± 0.09 mm in controls, 0.81 ± 0.10 mm in sham, 0.62 ± 0.08 mm in SAH-Survived, and 0.41 ± 0.07 mm in SAH-Nonsurvivor animals (Kruskal–Wallis *p* < 0.001), and the wall-to-lumen ratio increased correspondingly (0.18 ± 0.03 in controls vs. 0.42 ± 0.06 in SAH-Nonsurvivor, *p* < 0.001), confirming the qualitative impression of marked vasoconstriction. Perivascular sympathetic nerve plexuses in these rabbits showed degeneration (loss of normal axonal staining), whereas control animals had normal-appearing renal nerve fibers. Figure 4 shows renal artery vasospasm and Figure 5 demonstrates degeneration of the perivascular nerve plexus around it in an SAH subject. Additionally, SAH-Nonsurvivor kidneys showed small foci of intraparenchymal hemorrhages (likely from rupture of stress-weakened microvessels), whereas no hemorrhages were seen in controls.

*SAH-Nonsurvivor Subgroup:* In the SAH-Nonsurvivor subgroup, renal damage was extensive and universal. Grossly, the kidneys were swollen and displayed a mottled cortical surface. Microscopically, nearly every glomerulus was either completely atrophic or showed ghost-like morphology. The atrophic glomerulus density reached 98 ± 11 per cubic millimeter in these rabbits, essentially indicating that almost all glomeruli in each observed area were destroyed or nonfunctional. The few glomeruli that retained normal structure were typically small and isolated, suggesting they were residual remnants in less affected regions. Figure 6 and Figure 7 demonstrate the severe glomerular degeneration and presence of apoptotic tubular cells in an SAH-Nonsurvivor rabbit kidney. Many renal tubules contained proteinaceous casts, and there was clear evidence of acute tubular necrosis, likely secondary to severe ischemia and hypertension.

*Renal inflammation:* Alongside glomerular damage, SAH kidneys, especially in the SAH-Nonsurvivor subgroup, showed interstitial inflammation. We noted mononuclear cell infiltration (macrophages and lymphocytes) around glomeruli and blood vessels. Some glomeruli had crescents or fibrinoid necrosis, hinting that immune-mediated injury might be contributing (potentially triggered by damage-associated molecular patterns released after severe brain injury) [15,16]. This aspect was not quantitatively measured; it is consistent with the broader literature on inflammatory responses in severely brain-injured organ donors, which we cite as context rather than as a finding of the present study.

### 3.3. Correlation Between Vagal CAP Amplitude and Renal Parameters

Our data show a clear inverse relationship: across all individual animals, vagal CAP amplitude exhibited a strong negative correlation with atrophic glomerulus density (Spearman ρ = −0.89, bootstrap 95% CI −0.96 to −0.73, *p* < 0.001). Because the sample size was insufficient for stable multivariable modeling, the exploratory multivariable regression has been removed from the main text and is now reported only in the Supplementary Material (Supplementary Table S1); its coefficients are not interpreted here and carry no inferential weight. The primary correlation and the descriptive amplitude observation are summarized in Table 2.

Among SAH animals, those with vagal CAP amplitude around 1.0 mV (the survivors) retained approximately 50–60% normal glomeruli, whereas animals with vagal CAP amplitude near 0.3 mV (the non-survivors) had less than 10% normal glomeruli, with more than 90% categorized as atrophic. The value of 0.30 mV is not a threshold in any analytic sense. It is simply the upper bound of the amplitudes recorded in the four SAH-Nonsurvivor animals, each of which had more than 60% atrophic glomeruli. It was read off the same 19 observations from which every other result in this paper is drawn, was not derived by ROC analysis, has no associated sensitivity, specificity, or predictive value, and was not tested in any independent sample. It therefore cannot discriminate between animals, cannot be applied prospectively, and must not be used or cited as a cut-off. We report it purely as a descriptive, hypothesis-generating observation about the range of values we happened to record. Conversely, animals with vagal CAP amplitudes above 1.0 mV demonstrated minimal glomerular injury.

We also evaluated blood pressure in relation to these measures. Mean arterial pressure showed a positive correlation with atrophic glomerulus density, consistent with pressure-mediated kidney injury. The exploratory sensitivity analysis in Supplementary Table S1 is not interpreted further here, and we make no claim about the relative contributions of vagal CAP amplitude and blood pressure. Whether vagal dysfunction is linked to renal injury through processes beyond blood-pressure elevation, such as inflammation, cannot be determined from these data and is raised only as a hypothesis for future study.

## 4. Discussion

In this experimental study, electrophysiological vagal nerve dysfunction was strongly associated with renal histopathological injury in an experimental rabbit model of severe SAH. SAH produced clinical and hemodynamic findings consistent with marked autonomic imbalance, including sustained blood-pressure elevation, tachyarrhythmias, and neurogenic pulmonary edema. These changes were accompanied by histologic degeneration of vagal nerve fibers and parasympathetic ganglion neurons. We use the term “autonomic imbalance” rather than “autonomic storm” because sympathetic activity was not directly measured by plasma or tissue catecholamines or microneurography. Vagal impairment, quantified by reduced CAP amplitude, was closely correlated with the severity of glomerular loss. Animals with relatively preserved vagal signals showed better renal structural preservation, whereas those with nearly abolished vagal responses had severe glomerular injury. We make no claim about whether vagal CAP amplitude tracks glomerular injury more closely than mean arterial pressure does. The sample is far too small to rank the two, and the exploratory regression that addressed this question has been moved to the Supplementary Material and is not interpreted here. To our knowledge, no prior experimental study has directly linked vagal electrophysiological metrics with renal structural injury after severe SAH. Because this is a small, single-time-point, association-level study, these observations are best interpreted as preliminary and hypothesis-generating.

Our data are consistent with the hypothesis that reduced vagal integrity is associated with sustained sympathetic overactivity following acute brain injury, although the present design cannot establish that vagal injury causes this overactivity. Subarachnoid hemorrhage induces a robust and prolonged activation of the central sympathetic nervous system; for instance, total-body norepinephrine spillover in SAH patients increased approximately threefold within 48 h of hemorrhage and remained elevated over the first week after insult [9,17]. In parallel, acute brain injury, including SAH, disrupts parasympathetic regulation, diminishing vagal inhibitory tone and facilitating unchecked sympathetic discharge from hypothalamic and medullary centers [10,18]. In our SAH rabbits, sustained hypertension, tachycardia, and cardiac arrhythmias were observed; we interpret these as compatible with, but not evidence of, a catecholamine surge, since no catecholamine or sympathetic nerve activity measurements were made. The combination of reduced vagal inhibitory signaling and excessive sympathetic drive is compatible with a plausible pathophysiological link between brainstem injury after SAH and peripheral organ damage, including renal ischemia and glomerular loss.

Our observation that vagal CAP amplitude markedly dropped in animals showing the highest blood pressures and poorest outcomes aligns with prior experimental data. In an SAH rabbit model, degeneration of the vagal nodose ganglion was associated with renal artery vasospasm (vasospasm index increased from ~1.9 in sham to ~2.3 in SAH) and systemic hypertension, with animals developing pressures >122 mmHg (10). These findings are consistent with an association between parasympathetic (vagal) injury and neurogenic hypertension with altered renal vascular tone after SAH. Our study expands on this by providing a direct functional correlate (reduced vagal CAP amplitude) and linking it to histopathological kidney injury (glomerular and tubular damage). Reduced vagal output may coexist with sympathetic predominance and systemic hypertension, and renal hemodynamics may shift accordingly: vasoconstriction of afferent arterioles, reduced renal perfusion, and increased susceptibility to ischemia. Given also that SAH triggers systemic inflammatory responses that may harm renal microvasculature, sympathovagal imbalance and inflammation may coexist in severe kidney injury [1,8].

Reduced electrophysiological vagal integrity is, in principle, compatible with attenuated anti-inflammatory signaling; however, this pathway was not directly measured, and our data cannot establish that it was engaged. The vagus nerve mediates the cholinergic anti-inflammatory pathway, which modulates immune responses through efferent signals to the spleen and other immune organs; this pathway reduces production of proinflammatory cytokines (e.g., TNF-α, IL-6) by immune cells such as macrophages, thereby limiting tissue inflammation and injury [19,20]. In the SAH-Nonsurvivor animals, where vagal CAP amplitude was severely diminished, we observed marked mononuclear leukocyte infiltration and both glomerular and tubulointerstitial damage on routine histology. Because we did not directly measure cytokines, splenic acetylcholine, or vagal efferent nerve activity, the contribution of the cholinergic anti-inflammatory pathway in our model remains inferential, and the inflammatory findings described here should be interpreted as inferential pending direct biochemical confirmation. Clinical data support this mechanism: in aneurysmal SAH patients, higher admission hs-CRP levels were independently associated with development of AKI (odds ratio 1.2 per mg/L increase in hs-CRP) [1,21]. These clinical observations are consistent with a role for inflammation in SAH-related kidney injury, but they do not establish that reduced vagal anti-inflammatory tone produced a cytokine surge in our model, which we did not measure. We hypothesize that inflammatory injury acts in concert with the hemodynamic insult (hypertension, vasospasm) to produce the severe renal damage we observed; because no cytokine, catecholamine, or autonomic recordings were obtained, this remains an untested hypothesis rather than a conclusion supported by our data.

Such vasoconstriction may shunt blood flow away from glomeruli, inducing ischemia and predisposing to glomerular collapse, although renal blood flow was not measured here. Severe acute brain injury has separately been reported to provoke an inflammatory response in the kidney, including leukocyte infiltration and release of proinflammatory cytokines [22,23,24,25]. We cite this literature as background only; none of these mediators were measured in the present study.

One hypothesis compatible with these observations is that sympathetically mediated vasoconstriction, hemodynamic instability, and an inflammatory or coagulation-related cascade converged to produce widespread glomerular injury in our SAH-Nonsurvivor rabbits. The morphology we observed (glomerular collapse, ghost-like glomeruli, tubular necrosis, proteinaceous casts) is not inconsistent with such a multifactorial mechanism, but histologic appearance cannot identify a mechanism, and we measured none of the mediators involved. This account is offered as a hypothesis to be tested, not as an interpretation supported by the present data.

Because no interventional manipulation of vagal activity was performed, our data cannot speak to whether maintaining vagal activity would attenuate injury; we observed only that preserved vagal CAP amplitude was associated with less severe glomerular injury. In our SAH rabbits, those with higher vagal CAP amplitude showed less extensive glomerular injury, whereas those with markedly reduced amplitudes showed near-total glomerular atrophy and sclerosis. This pattern mirrors preclinical models in which vagus-nerve stimulation markedly reduces kidney inflammation, oxidative stress, and structural damage after ischemia/reperfusion injury [11,26]. Whether the association we observed shares any mechanism with those interventional models is untested.

This study neither modelled formally diagnosed brain death nor examined transplantation, graft function, or donor management, and we therefore do not develop these themes further. We note only that whether any autonomic measure relates to renal vulnerability in the donor setting is unknown and would have to be established in studies designed for that purpose [14,20].

The kidney is vulnerable to hemodynamic instability, and the sustained pressure elevation we recorded may have contributed to the glomerular and vascular injury seen in this model [27,28,29]. Our data cannot inform blood-pressure targets in any clinical setting, including donor management, and we make no recommendation on that basis.

### Methodological Considerations and Study Limitations

This was an animal model that used rabbits with experimentally induced SAH. Although this model reproduces several features of severe acute brain injury, including sympathetic surges and peripheral organ injury, progression was faster than in humans (1–2 weeks vs. several days) [30,31,32]. Critically, this model does not reproduce the formal clinical diagnosis of brain death. We did not perform standardized apnea testing, electroencephalography, or ancillary blood-flow studies, and we did not demonstrate irreversible cessation of all brainstem function. We therefore describe our most severely affected animals as SAH-Nonsurvivors with terminal brainstem dysfunction, not as brain-dead, and we have removed brain-death terminology from the central claims of this study. This is an important conceptual limitation that constrains extrapolation of our findings to the brain-dead organ donor. Vagal CAP amplitude (compound action potential amplitude) was used as an aggregate endpoint metric. A low amplitude likely reflects vagal axonal loss or impaired neurotransmission and correlated well with histological neuron counts, but it provides no temporal information. Continuous autonomic monitoring, such as heart-rate variability (HRV), could clarify the timing of reduced vagal CAP amplitude relative to renal injury [33,34]. HRV was not analyzed in the present study because high-fidelity ECG sampling was unavailable on a daily basis, and we therefore could not derive validated time- or frequency-domain HRV indices. Likewise, plasma or tissue catecholamines, microneurography-based muscle sympathetic nerve activity, and cytokine panels (TNF-α, IL-6, MCP-1) were not measured; hence, our inferences about “sympathetic surge” and “cholinergic anti-inflammatory pathway” involvement are based on hemodynamic and histologic surrogates rather than on direct biochemical confirmation, and should be tested in future studies that include simultaneous catecholamine and cytokine sampling, vagotomy/VNS interventions, and continuous HRV monitoring. Because tissue analysis was performed at a single time point, our results show association rather than proven causality. Several non-causal explanations must be considered explicitly: (i) global ischemic injury from severe SAH could damage vagal pathways and renal tissue in parallel; (ii) overall SAH severity could act as a common upstream determinant of both reduced vagal CAP amplitude and glomerular injury; (iii) systemic hemodynamic instability or terminal circulatory collapse could independently drive both findings; and (iv) in the SAH-Nonsurvivor subgroup, post-mortem effects could contribute to the lowest recorded CAP amplitudes. Without mechanistic interventions (vagotomy, vagus-nerve stimulation, pharmacological modulation) or longitudinal physiological assessment, the present design cannot determine whether reduced vagal CAP amplitude contributes to renal injury or merely reflects disease severity. In addition, intracranial pressure was not continuously recorded in individual animals; brain injury severity was inferred from established model parameters and clinical signs of brainstem dysfunction.

For early-death animals, tissues were harvested as soon as possible after death (median post-mortem interval 18 min, range 5–30 min); no validation of vagal CAP stability across this 5–30 min window was performed in the present study, and we did not conduct a post-mortem time-course experiment or any other test of amplitude stability in this preparation. Vagal compound action potential amplitudes are known to attenuate with progressively longer ischemic intervals. We therefore cannot exclude that post-mortem deterioration contributed to the reduced amplitudes recorded in SAH-Nonsurvivor animals, and the size of any such contribution is unknown. Because the SAH-Nonsurvivor subgroup differed from all other cohorts both in disease severity and in recording conditions, these two influences are confounded and cannot be separated in the present design. All subgroup comparisons involving SAH-Nonsurvivors must therefore be regarded as exploratory and potentially biased, and the reported amplitudes in this subgroup should be treated as an upper bound on measurement fidelity rather than as clean physiologic values. A dedicated post-mortem time-course experiment, or intravital recording before circulatory arrest, would be required to resolve this and is a priority for future work.

Nonetheless, we acknowledge this as a methodological limitation. Further interventional studies using pharmacological or electrical vagus nerve stimulation are needed to determine causality. Inter-individual variation was considerable, paralleling the clinical heterogeneity of SAH. Differences in vascular anatomy or genetic background may have influenced severity, though statistical trends remained robust. Direct vagal CAP amplitude measurement in humans would be invasive, but non-invasive autonomic surrogates could serve as translational tools. HRV, particularly its high-frequency component, is a validated correlate of vagal tone in physiologic studies. Reduced HRV after acute brain injury has been linked to worse outcomes and higher mortality. Yet HRV interpretation has methodological limits: the assumption that high-frequency HRV purely reflects parasympathetic tone may not hold under unstable conditions [33,35].

**Translational Statement:** This exploratory experimental study examined whether an electrophysiological index of vagal nerve integrity (vagal CAP amplitude) is associated with the degree of glomerular injury after severe subarachnoid hemorrhage (SAH) in rabbits. The study describes a statistical association in a small animal cohort; it does not establish causality, does not model formally diagnosed brain death, and does not evaluate transplantation or graft function. Any translational implications for donor organ assessment are speculative and are raised only as hypotheses for future, adequately powered and mechanistically controlled studies.

## 5. Conclusions

Our findings relate to the broader question of how acute brain injury affects peripheral organs, particularly the kidney. The downstream organ effects of severe brain injury are thought to arise partly from physiological, inflammatory, and hemodynamic disturbances [27,36]; our data add an autonomic (vagal) structural correlate to this picture in an experimental SAH model. Clinical donor-management strategies are outside the scope of this experimental study and are not addressed.

Within the limits of a small, exploratory, association-level study, our data suggest that vagal nerve integrity may be related to the severity of renal structural injury after severe SAH. We do not claim that vagal CAP amplitude is a validated marker of kidney quality, nor that the autonomic nervous system is an established determinant of renal outcome; these remaining hypotheses require confirmation. Reduced vagal activity was associated with severe glomerular injury, supporting further study of whether autonomic imbalance is mechanistically involved in renal injury following acute brain damage; causality cannot be established from the present design. Any clinical or donor-management implications are necessarily speculative at this stage and would require, at minimum, larger adequately powered studies, mechanistic (interventional) confirmation, and validation in a model that reproduces formally diagnosed brain death.

In summary, this exploratory study reports an inverse association between vagal CAP amplitude and glomerular injury in a small rabbit model of severe SAH. The principal contribution is to motivate larger, adequately powered, and mechanistically controlled studies of the autonomic nervous system in acute brain injury and renal outcome. No clinical or donor-management recommendation can be drawn from the present data.

## Figures and Tables

**Figure 1:**
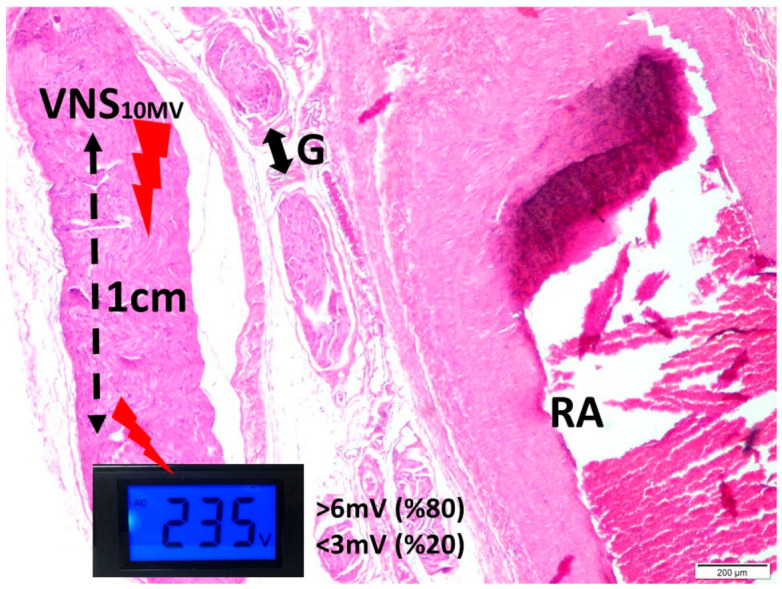
Schematic of the experimental set-up and representative histopathology. Diagram of vagal nerve stimulation: a bipolar stimulating electrode is placed on the cervical vagus and the evoked compound action potential is recorded distally; peak-to-peak amplitude (mV) defines vagal CAP amplitude. Hematoxylin–eosin section of the renal hilum showing the renal artery (RA) and adjacent perirenal parasympathetic ganglion (G; arrow). Scale bars as indicated on individual panels. The annotation “VNS 10 mV” on the stimulator display is a representative screenshot showing intensity in millivolts (mV), not volts or megavolts; as stated in the Methods, the stimulus was not fixed at this value but was individually set for each animal at approximately 20% above the level producing a maximal response. RA = renal artery; CAP = compound action potential, VNS: Vagal Nerve Stimulation.

**Figure 2:**
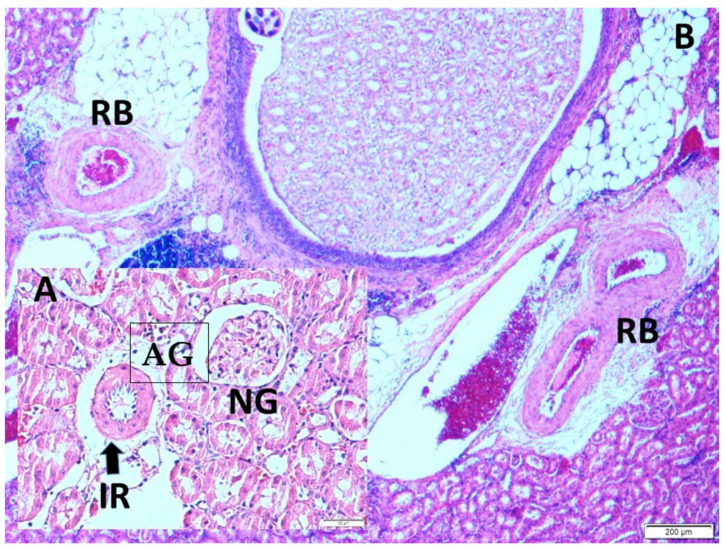
Glomerular histology. (**A**) Control kidney: well-perfused glomerulus with open capillary loops adjacent to a normal interlobular artery. (**B**) SAH rabbit kidney: normal (NG) and atrophic (AG) glomeruli within the same field. Atrophic glomeruli show capillary tuft collapse, basement-membrane wrinkling, and peri-glomerular fibrosis. Hematoxylin and eosin; original magnification ×200. Image abbreviations: DG, degenerated (“ghost”) glomerulus. Where panel-level coloration differs between panels A and B, this reflects regional differences in tissue preservation rather than a different histochemical stain. AG = atrophic glomerulus; NG = normal glomerulus, IR: Intrarenal artery, RB. Examined Renal Branch).

**Figure 3:**
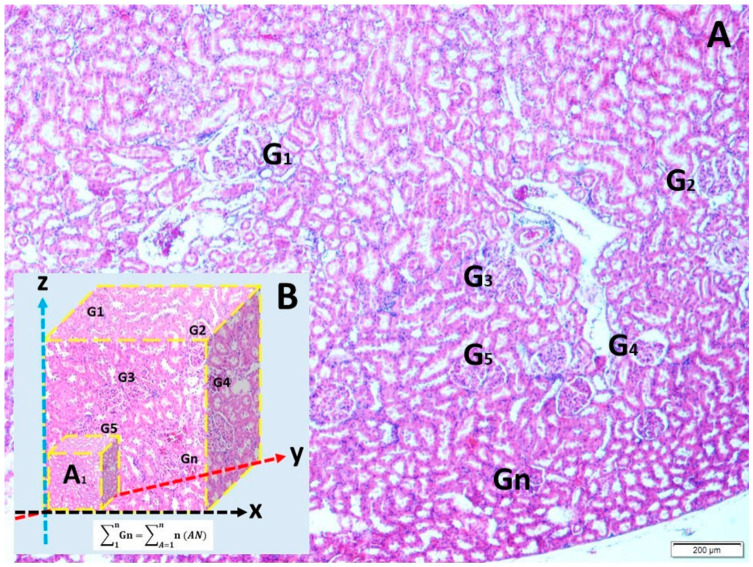
Stereologic method for glomerular density. Sequential 5 µm sections were obtained; on systematically sampled section pairs the optical disector was applied (**A**), and glomerular profiles present in the reference but not in the look-up section were counted (ΣQ^−^). Numerical density (Nv, glomeruli/mm^3^) was calculated as Nv = ΣQ^−^/(t × A-frame), where t is section thickness and A-frame is the counting-frame area. Atrophic and non-atrophic glomeruli were distinguished by predefined histologic criteria (**B**). G1, G2, G3, …… Gn: number of glomerules, 1 to n).

**Figure 4:**
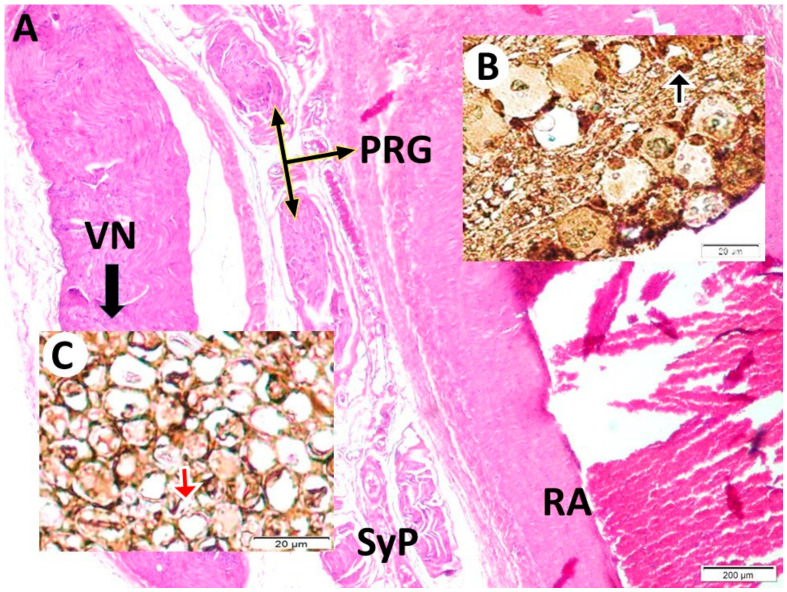
Renal vascular and neural injury in an SAH rabbit (**A**). Renal artery showing wall thickening and luminal narrowing. Degenerated perirenal parasympathetic ganglion neurons represented with black arrow with shrunken hyperchromatic neurons and perineuronal halos (PRG in (**B**)). Disorganized peri-arterial sympathetic plexus (SyP in (**A**)) with reduced axonal staining in figure (**C**). Vagal trunk with axonal fragmentation consistent with Wallerian degeneration showned with red arrow in (**C**). Hematoxylin and eosin and neuron-specific enolase immunostaining; original magnification ×100–×400. Image abbreviations: VN, vagal nerve; RA, renal artery; SyP, sympathetic plexus; PRG (sometimes labeled DRPG), perirenal/degenerated renal parasympathetic ganglion; arrows indicate the structures named in each panel.

**Figure 5:**
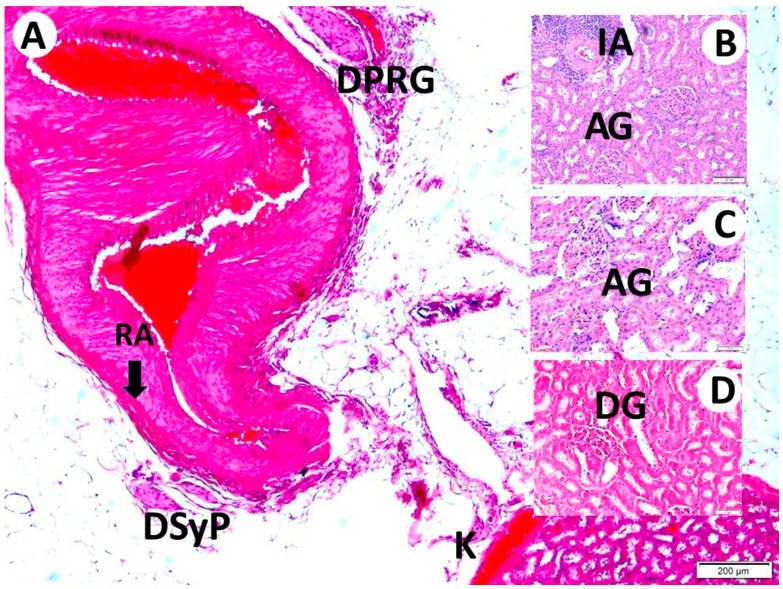
Additional renal histology in an SAH rabbit. Constricted renal artery with narrowed lumen (RA, severely degenerated perirenal parasympathetic ganglion (DPRG) and degenerated sympathetic plexus (DSyP) and kiney tissue (K) in (**A**). Intrarenal artery surrounded by mononuclear (lymphoid) infiltrate, with adjacent edematous and atrophic glomeruli (AG) in (**B**). (**C**) shows fewer atrophic glomeruli (AG) in specimens with fewer atrophic neurons, whereas (**D**) shows more atrophic glomeruli in specimens with a greater number of atrophic neurons. Hematoxylin and eosin; original magnification ×200. Image abbreviations: AG, atrophic glomerulus; G, glomerulus (non-atrophic); IA, intrarenal artery; K, kidney parenchyma; DPRG, degenerated perirenal parasympathetic ganglion; DSyP, degenerated sympathetic plexus; DG, degenerated glomerulus.

**Figure 6:**
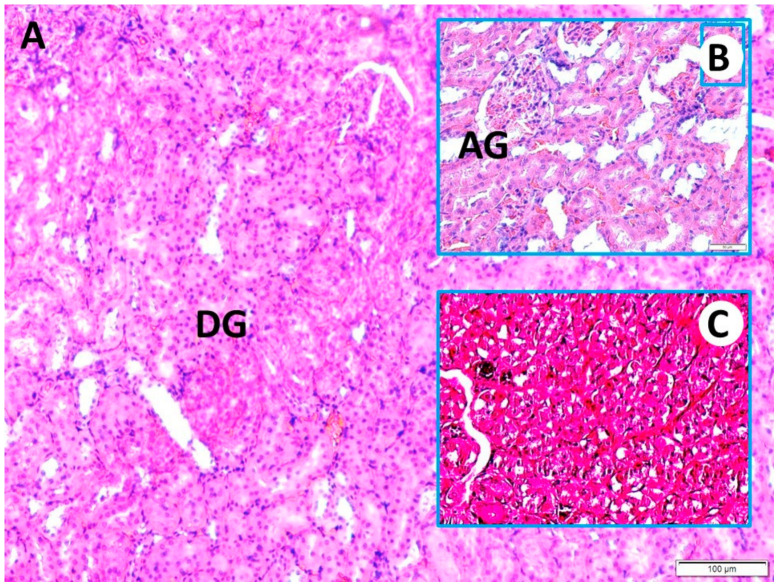
Severe renal injury in an SAH-Nonsurvivor rabbit in (**A**). Parenchymal hemorrhagic edema with ghost glomeruli (collapsed acellular tuft outlines) and degenerated glomeruli (DG) (in **A**). Adjacent tubules show proteinaceous casts and acute tubular necrosis in atrophic glomerulus (AG) in (**B**). Totally atrophic glomeruli (AG) and hemorrhaged kidney tissue is seen in (**C**). Hematoxylin and eosin; original magnification ×200.

**Figure 7:**
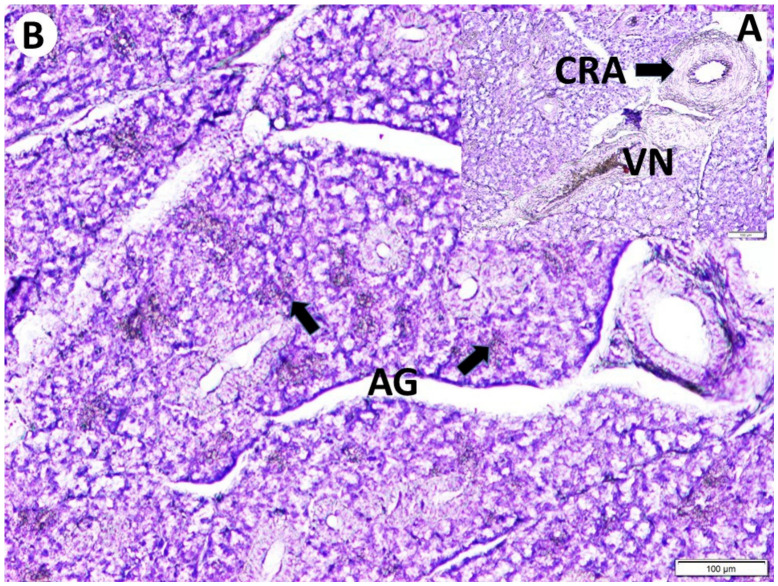
Histopathological appearance of constricted renal artery (CRA), injured vagal nerve (VN) fibers, atrophic glomeruli (AG) in (**A**), and apoptotic renal tubular cells (Darkened cells in base) in atrophic glomeruli (AG) in (**B**), in an SAH-Nonsurvivor rabbit. Hematoxylin and eosin; original magnification ×200–×400.

**Table 1:** Hemodynamic (MAP), electrophysiologic, and renal histopathologic parameters.

Group	Mean Arterial Pressure (mmHg)	Vagal CAP Amplitude (mV)	Degenerated Neurons in Renal Parasympathetic Ganglia (per mm^3^)	Atrophic Glomeruli (per mm^3^)
Control, *n* = 5	99 ± 7	1.42 ± 0.36	6 ± 2	4 ± 1
Sham, *n* = 5	106 ± 8	1.23 ± 0.29	21 ± 5	12 ± 3
SAH-Survived, *n* = 5	117 ± 13	0.95 ± 0.12	25 ± 7	56 ± 8
SAH-Nonsurvivor, *n* = 4	126 ± 8 *	0.34 ± 0.11	98 ± 19	98 ± 11

Values are presented as mean ± SD. MAP was compared among groups using one-way ANOVA with Tukey’s post hoc test; the most prominent between-group difference is marked with an asterisk (* *p* < 0.05 vs. control). Histologic count variables, including degenerated neuronal density and atrophic glomerular density, were compared using the Kruskal–Wallis test with Dunn’s post hoc test. For atrophic glomerular density, significant pairwise differences were found for SAH-Survived vs. control (*p* < 0.01), SAH-Nonsurvivor vs. control (*p* < 0.001), SAH-Nonsurvivor vs. sham (*p* < 0.001), and SAH-Nonsurvivor vs. SAH-Survived (*p* < 0.01). For degenerated neuronal density, SAH-Survived vs. sham (*p* < 0.05), SAH-Nonsurvivor vs. control (*p* < 0.001), and SAH-Nonsurvivor vs. SAH-Survived (*p* < 0.01) were significant. For vagal CAP amplitude, SAH-Survived vs. control (*p* < 0.01), SAH-Nonsurvivor vs. control (*p* < 0.001), and SAH-Nonsurvivor vs. SAH-Survived (*p* < 0.01) were significant. Pairwise *p*-values for the histologic count variables (degenerated neuronal density, atrophic glomerular density) are Dunn’s adjusted post hoc values. Pairwise comparisons for MAP and vagal CAP amplitude were performed using Tukey’s post hoc test following one-way ANOVA, where parametric assumptions were considered reasonable. MAP, mean arterial pressure; CAP, compound action potential; mV, millivolt; mm^3^, cubic millimeter; SAH, subarachnoid hemorrhage; SD, standard deviation.

**Table 2:** Correlation between vagal CAP amplitude and glomerular injury, with a descriptive observation on the range of amplitudes recorded in non-surviving animals.

Analysis Type	Variables Compared	Statistic	Value
Correlation	Vagal CAP amplitude vs. atrophic glomerulus density	Spearman ρ, 95% CI, *p* value	−0.89; 95% CI −0.96 to −0.73; *p* < 0.001
Descriptive observation	Vagal CAP amplitude vs. severe glomerular atrophy	Highest amplitude recorded in a non-surviving animal (descriptive only; not a tested cut-off)	0.30 mV
		Atrophic glomeruli	>60%
		Survival	In this dataset, amplitudes below 0.3 mV occurred only in non-survivors (*n* = 4); not evaluated as a predictor

ρ, Spearman correlation coefficient; mV, millivolt; CAP, compound action potential; n, number. The amplitude value in the second row is descriptive, not a derived threshold: it is simply the upper bound of the amplitudes recorded in the four non-surviving animals. It was read off the same small dataset (*n* = 19), was not derived by ROC analysis, has no associated sensitivity or specificity, and was not tested in any independent sample; it therefore has no discriminative or predictive value and must not be used or cited as a cut-off. The exploratory multivariable regression previously shown here has been moved to Supplementary Table S1.

## Data Availability

The datasets generated and analyzed during the current study are available from the corresponding author upon reasonable request.

## Supplementary Materials

The following supporting information can be downloaded at https://www.mdpi.com/article/10.3390/jcm15156056/s1. Figure S1. Kaplan–Meier survival, Figure S2. TUNEL-positive neurons per ganglion section, Figure S3. Daily mean arterial pressure, Figure S4. Group-wise atrophic glomerular density and the vagal CAP amplitude versus atrophic glomerular density scatterplot, Table S1. Exploratory multivariable regression, presented as a sensitivity analysis only and not interpreted in the main text.
