# Supplementary material for: Reduced Vagal Compound Action Potential Amplitude Is Associated with Glomerular Injury in an Experimental Rabbit Model of Severe Subarachnoid Hemorrhage
Source: J Clin Med. 2026 Aug 4;15(15):6056. doi: 10.3390/jcm15156056 (PMC13466770; doi:10.3390/jcm15156056)
Supplement: Supplementary file 1 [file jcm-15-06056-s001.zip › Table S1.pdf]

Supplementary Material

Table S1. Exploratory multivariable linear regression with atrophic glomerular density (per mm^3) as the dependent variable and vagal CAP amplitude and mean arterial pressure (MAP) as predictors (n = 19). Presented as a sensitivity analysis only; not interpreted in the main text, and the coefficients are not treated as effect estimates.

**Predictor**	**Standardized β**	**p-value**
Vagal CAP amplitude (mV)	−0.75	< 0.01
Mean arterial pressure (mmHg)	0.45	0.07

Abbreviations: CAP, compound action potential; MAP, mean arterial pressure; mV, millivolt; mm^3, cubic millimetre.

Note. With only 19 observations for two predictors, formal residual diagnostics, variance-inflation assessment, the coefficient of determination (R^2), and coefficient confidence intervals would be unstable and were therefore not computed, consistent with the main-text Statistical Analysis section. Only the standardized coefficients and their p-values are reported, solely for transparency; they are not interpreted as effect estimates and are not used to support any statement in the manuscript. The primary analysis reported in the main text is the Spearman rank correlation (ρ = −0.89, 95% CI −0.96 to −0.73; 2000 bootstrap resamples).
